# Long-term labour market outcomes of anorexia nervosa – the Northern Finland birth cohort 1986

**DOI:** 10.1007/s00127-025-02983-4

**Published:** 2025-08-26

**Authors:** Tuomas Majuri, Emmi Wilén, Sanna Huikari, Marko Korhonen

**Affiliations:** 1 Research Unit of Population Health, University of Oulu, Oulu, Finland; 2 Medical Research Center Oulu, Oulu University Hospital and University of Oulu, Oulu, Finland; 3 Terveystalo Occupational Healthcare, Oulu, Finland; 4 Department of Economics, Accounting and Finance, University of Oulu, Oulu, Finland

**Keywords:** Anorexia, Cohort study, Income, Unemployment, Sickness benefits

## Abstract

**Purpose:**

Anorexia nervosa (AN) is one of the most severe mental disorders in adolescence, yet it generally has a favourable long-term prognosis. However, evidence on the long-term labour market outcomes of AN is scarce and has several key limitations. This study aimed to provide a comprehensive assessment of the current long-term labour market outcomes of AN, examining unemployment, sickness absences, and income up to age 33.

**Methods:**

Utilizing data from the Northern Finland Birth Cohort 1986, linked to national registers, we compared unemployment days, sickness absences, and income between individuals with AN (*n* = 165) and the rest of the cohort (control group, CG; *n* = 4884) using the Welch’s t-test and two-part regression models. The analyses were stratified by sex.

**Results:**

Compared to the CG, both males and females with AN had lower cumulative income in 2011–2016. AN was associated with a greater number of unemployment days among both males and females who had any unemployment days. No significant differences in sick days in 2011–2019 were found between individuals with AN and the CG in either sex.

**Conclusion:**

This study highlights the association between AN and poor labour market outcomes, particularly regarding income accumulation. More effective strategies are needed to identify and support individuals with AN, especially males, who tend to experience worse outcomes. Increasing awareness of AN among students and in workplaces, enhancing collaboration between services, and implementing early, cost-effective interventions are essential for improving labour market prospects.

**Supplementary Information:**

The online version contains supplementary material available at https://doi.org/10.1007/s00127-025-02983-4.

## Introduction

Anorexia nervosa (AN) is a severe mental disorder that typically emerges during adolescence, but its long-term prognosis varies, with some individuals achieving recovery while others continue to experience chronic symptoms [1].

Most studies on AN have primarily focused on clinical outcomes, such as symptomatology and recovery rates [1], while neglecting occupational aspects such as employment, sickness benefits, and income in their evaluations of recovery [2]. This omission has contributed to a lack of data on labour market outcomes for individuals with AN. This research gap is particularly evident when compared to studies on other severe mental health disorders, where the labour market perspective is often considered part of the recovery criteria [3, 4].

Evidence on the long-term labour market outcomes of AN is scarce and has key limitations. First, most findings are based on studies published 10–19 years ago, involving individuals born in the 1960 s and 1970 s [5–7]. Generalizing these results to the present is challenging, as working life and psychiatric services have undergone significant changes over the past decades. The new ways of working, new technologies, and the rise of digitalization have created both opportunities and challenges for employees with mental health disorders [8]. Since the 1990 s, Finland has followed the international trend of reducing psychiatric inpatient services [9, 10], affecting the identification and prevalence of various mental health disorders. According to a recent meta-analysis, the prevalence of AN is increasing but may still be underestimated [11]. In Finland, half of individuals with AN remain undetected by the healthcare system [12].

Secondly, sex differences in mental health [13], employment opportunities [14], and work-life courses in general population samples [15] have been documented. Previous studies have analysed labour market outcomes of AN exclusively in females, entirely overlooking males and potential sex differences [5–7]. Males with eating disorders are often excluded from research due to being considered ‘atypical’ cases [16]. Additionally, recent studies have shown that AN is underdiagnosed, particularly among males [17]. Consequently, a general lack of understanding of AN in males has prompted recommendations for sex-specific analyses [16, 17]. Neglecting sex differences could obscure significant findings, introduce bias, and limit the generalizability of the conclusions.

Thirdly, the few existing studies addressing the long-term labour market outcomes of AN have typically examined them as part of overall outcomes [5–7]. None have specifically focused on labour market outcomes — that is, how individuals with AN develop their careers and adapt to the workforce. Furthermore, these studies have assessed labour market outcomes primarily using questionnaire-based data on education, employment, and sickness benefits [5–7], overlooking register data and some important factors such as income accumulation. A Swedish study found that among individuals with AN, 9–14 years after hospital admission, 58% had completed basic or post-secondary education, 13% were still studying, 58% were employed, and 21% were dependent on disability pension or other sickness benefits [5]. Another Swedish study found that 25% of individuals with adolescent-onset AN had no paid employment 18 years after the disorder’s onset [6]. A Finnish study found that after a 10-year follow-up, women with AN did not significantly differ from their unaffected peers in terms of completing university degrees, the number of sickness absences during the past year, or unemployment and disability pensions [7]. However, they were more often still studying [7].

Updated information on the long-term labour market outcomes would be valuable for raising awareness and developing interventions for individuals with AN. There is a need for studies that consider current diagnostic practices, sex differences, changes in working life, and focus on the long-term labour market outcomes of AN. This study aimed to provide a comprehensive assessment of the current long-term labour market outcomes of AN examining unemployment, sickness absences, and income. Given the established sex differences in labour market outcomes associated with mental disorders [18, 19], we stratified our analyses by sex. The data included prospectively collected national-level register data linked to general population birth cohort with follow-up until the age of 33 years. This cut-off was chosen based on data availability and to allow sufficient time for participants to complete their education and establish themselves in the labour market.

## Method

### Sample

The study was based on the Northern Finland Birth Cohort 1986 (NFBC1986) [20], which is an unselected, general population sample based on 9432 live-born children with an expected date of birth between July 1 st, 1985 – June 30th, 1986, in the provinces of Oulu and Lapland [21]. The cohort members have been followed up with data collection at different ages and with individual-level linkage to registered data from various highly reliable Finnish national registers.

In the NFBC1986 16-year follow-up study, questionnaire data were received from 7182 (76%) and clinical data from 6795 (72%) adolescents. In this study, we utilized data on physical health and nutrition at the age of 16. We identified cohort members with information on BMI (clinical examination data) and self-perceived weight (questionnaire data) at age 16. These individuals comprised the final study sample (*n* = 5049).

### Case detection

Because the diagnostic criteria for AN remain insufficiently inclusive of sex diversity [16], and half of the cases are not detected by the healthcare system [12], we used a broad definition for AN. Applying ICD-10 criteria for atypical AN (F50.1), we defined AN as BMI ≤ 17.5, coupled with a perception of being an appropriate weight or overweight despite being significantly underweight (questionnaire data from the NFBC1986 16-year follow-up). The age cut-off of 16 years for AN was based on previous Finnish studies reporting peak [22] and mean [7, 12] onset at that age, and was chosen to capture cases before major educational and occupational transitions.

Based on these criteria, we identified 92 males and 73 females with AN in the sample. For comparison purposes, the remaining cohort members (2334 males and 2550 females) were used as controls (control group, CG).

### Measures

#### Background characteristics

The following variables were used to characterise the sample. The same variables were used as covariates in the adjusted analyses.

The educational level of the participants’ parents was retrieved from the NFBC1986 16-year follow-up questionnaire. Information on the participants’ highest attained educational level was gathered from the register of Statistics Finland (until 2016) [23]. Both participants’ and their parents’ educational levels were classified according to the International Standard Classification of Education [24], using the categories: basic education (9 years or less), secondary education (9 to 12 years), and tertiary education (more than 12 years). For participants, tertiary education was further divided into lower tertiary (12 to 16 years) and higher tertiary education (more than 16 years). Different categorizations were applied to participants and their parents due to the significant increase in the proportion of individuals with higher education between the parents’ generation and that of the cohort members. Information on family type, categorized as a family with two biological parents, or other was based on the NFBC1986 16-year follow-up questionnaire data. Information on the participants’ grade point average (GPA) of theoretical subjects (range 4–10) when leaving compulsory education at the age of 16 years was gathered from the register of the Finnish national application system for upper secondary education [25]. The register of the Digital and Population Data Services Agency (until 2016) was used to obtain information on marital status (married/in a registered partnership vs. not) [26]. Register data on socioeconomic status (SES) at the age of 30 years was used [23]. SES included the following categories: farmers, entrepreneurs, upper white collar, lower white collar, manual workers, students, pensioners, and others, mostly unemployed and was classified as (1) white collar i.e., lower to upper white collar, (2) other categories.

#### Labour market outcomes

Data on unemployment days were obtained from the Finnish Centre for Pensions (FCP) registers [27], while data on sick leave days were retrieved from the registers of the Social Insurance Institution of Finland [28] and the FCP [27]. The sick leave data included all sickness allowance periods exceeding the standard 10-working-day waiting period. Unemployment and sick leave days were presented for the timespan 2011–2019, when individuals were aged 25 to 33 years.

Information on cohort members’ taxable cumulative income was collected from the Finnish Tax Administration register and was presented in 2016 euros [29]. The variable included annual wage and salary earnings, self-employment earnings, and social income transfers such as sick leave benefits. Cumulative income was presented for the period 2011–2016, when individuals were aged 25 to 30 years.

The decision to analyse labour market outcomes starting from 2011 was made to evaluate prognosis after most of the sample had entered the workforce. For all the persons born in Finland in 1985–1986, the employment rate surpassed 70% in 2011 [30]. The specific time periods used to analyse different labour market outcomes were determined by the availability of register data, with unemployment and sick leave days available until 2019, and cumulative income until 2016.

### Statistical analyses

#### Background characteristics

The background variables for the AN and the CG were calculated separately by sex using cross-tabulation (categorical variables) and means with standard deviations (continuous variables).

#### Labour market outcomes

The unemployment days (2011–2019), sick days (2011–2019), and cumulative income (2011–2016) were calculated using means with standard deviations. The Welch’s t-test was used to compare outcomes between the study groups.

To examine the association between AN and the extent of unemployment and sick days (both 2011–2019), we used two-part regression models. In both models, the first part used logistic regression to estimate the probability of having any unemployment or sick days, while the second part used zero-truncated negative binomial regression to model the number of days among individuals with non-zero values. These models were selected due to the count nature of the outcomes, their skewed distributions, and the presence of overdispersion. Model diagnostics (Akaike Information Criterion, Bayesian Information Criterion, log-likelihood, and a likelihood-ratio test) confirmed the appropriateness of the negative binomial distribution in both cases.

To analyse cumulative income in 2011–2016, we applied a two-part regression model. A two-part model was used to account for the fraction of individuals not in the workforce. The first part used a multivariable logistic regression to estimate the probability of being employed. Given that our income variable encompasses both salary earnings and social income transfers, we applied the yearly income threshold of 12,000€ (9,844€ in 2016 euros) determined by the Finnish Immigration Service to classify individuals as working or not [31]. This threshold is based on basic social assistance amounts and it measures whether an individual has sufficient financial resources to reside in Finland. Individuals with average yearly income per period under the threshold were considered not working in that period. The second part employed ordinary least squares (OLS) regression with a logarithmic transformation of income to model cumulative income among those classified as working.

Numerous risk factors influencing labour market outcomes among individuals with mental disorders have been documented in the literature [32–34]. Based on these previous findings [32–34], we included the following covariates in all models: parents’ educational level as a proxy for childhood socioeconomic background; educational level and GPA as indicators of academic attainment and performance; marital status and family type to account for social and household context; and socioeconomic status to capture accumulated social advantage or disadvantage.

Odds ratios (ORs) with 95% confidence intervals (CIs) and regression coefficients with standard errors (SEs) were reported. *P*-values < 0.05 were considered statistically significant. All analyses were conducted using Stata version 16.0.

## Results

### Characteristics of the sample

Compared to the CG, individuals with AN had lower educational levels and parents’ educational levels (Online supplement 1). Females were more likely than males to have white-collar jobs, be married or in a registered partnership, and have higher average school grades and educational levels in both groups. Males in the CG more often had both parents compared to males with AN.

### Unemployment days

Males with AN had higher mean number of unemployment days in 2011–2019 (565 for AN and 330 for CG) compared to the CG (Table 1). For females, the corresponding mean numbers of unemployment days were 473 for AN and 355 for CG in 2011–2019, but the difference was not statistically significant.

**Table 1:** Differences in cumulative earnings (2011–2016) and unemployment and sick days (2011–2019) between AN and CG

		Male			
		Control group (*n* = 2334)		Anorexia (*n* = 92)	*p*-value
Cumulative income (€), M (SD)	2011–2016	156,050 (77,080)		123,410 (71,350)	*p* < 0.001
Unemployment days, M (SD)	2011–2019	330 (610)		565 (807)	*p* = 0.007
Sick days, M (SD)	2011–2019	19 (66)		24 (71)	*p* = 0.495

		Female			
		Control group (*n* = 2550)		Anorexia (*n* = 73)	*p*-value
Cumulative income (€), M (SD)	2011–2016	125,030 (58,650)		109,000 (49,020)	*p* = 0.008
Unemployment days, M (SD)	2011–2019	355 (556)		473 (646)	*p* = 0.125
Sick days, M (SD)	2011–2019	26 (80)		28 (70)	*p* = 0.818

*M* mean, *SD* standard deviation

The results of the regression model for unemployment days in 2011–2019 are presented in Table 2. AN was not associated with a higher probability of unemployment for either sex. However, AN was associated with a greater number of unemployment days among both males and females who had any unemployment days. Among males and females who experienced unemployment, those with AN had a 33% and 35% higher expected number of unemployment days, respectively, compared to the CG.

**Table 2:** Two-part regression model, unemployment days in 2011–2019

	Male				Female			
	Logit		Negative binomial		Logit		Negative binomial	
*Variable*	*OR*	*95% CI*	*Coefficient*	*SE*	*OR*	*95% CI*	*Coefficient*	*SE*
GPA	1.234***	1.113–1.368	−0.046	(0.036)	1.382***	1.243–1.537	−0.027	(0.033)
Lower tertiary education	0.556***	0.434–0.711	−0.060	(0.085)	0.971	0.786-1.200	−0.390***	(0.072)
Higher tertiary education	0.506***	0.369–0.695	−0.447***	(0.124)	0.458***	0.347–0.605	−0.459***	(0.088)
Parental secondary education	0.769	0.536–1.103	−0.001	(0.133)	1.070	0.735–1.558	−0.104	(0.102)
Parental tertiary education	0.938	0.626–1.405	−0.001	(0.151)	1.182	0.780–1.792	−0.111	(0.117)
Both parents	1.325**	1.073–1.636	−0.090	(0.068)	1.107	0.906–1.354	−0.044	(0.058)
Anorexia	0.742	0.483–1.141	0.287*	(0.141)	0.739	0.445–1.227	0.300*	(0.125)
White collar	2.340***	1.891–2.895	−0.555***	(0.087)	2.454***	2.044–2.945	−0.630***	(0.050)
Married	1.866***	1.554–2.242	−0.451***	(0.084)	1.232*	1.045–1.452	−0.023	(0.052)
ln alpha	0.215	*z-value* *5.75*			0.049	*z-value* *1.56*		
alpha	1.240				1.050			
N (total)	2426				2623			
N (unemployment days > 0)			1219				1537	

*OR* odds ratio, *CI* confidence interval, *SE* standard error, *GPA* grade point average

* *p* < 0.05, ** *p* < 0.01, *** *p* < 0.001

### Sick days

Males with AN had a mean number of 24 sick days in 2011–2019, compared to 19 in the CG (Table 1). For females, the mean number of sick days was 28 for AN, compared to 26 in the CG. The mean number of sick days did not differ significantly between groups for either sex.

The results of the regression model for sick days (2011–2019) are presented in Table 3. AN was not independently associated with either the probability of having sick days nor the number of sick days among males or females.

**Table 3:** Two-part regression model, sick days in 2011–2019

	Male				Female			
	Logit		Negative binomial		Logit		Negative binomial	
*Variable*	*OR*	*95% CI*	*Coefficient*	*SE*	*OR*	*95% CI*	*Coefficient*	*SE*
GPA	1.082	0.956–1.225	−0.013	(0.061)	1.227***	1.094–1.376	−0.134	(0.066)
Lower tertiary education	1.827***	1.323–2.523	−0.537**	(0.201)	0.994	0.788–1.255	−0.231	(0.140)
Higher tertiary education	2.044**	1.329–3.145	−0.573	(0.362)	1.719**	1.242–2.379	−0.408	(0.210)
Parental secondary education	0.745	0.480–1.156	0.262	(0.295)	1.452	0.974–2.164	0.062	(0.192)
Parental tertiary education	1.111	0.664–1.858	0.353	(0.347)	1.303	0.830–2.044	0.042	(0.217)
Both parents	1.074	0.837–1.379	−0.265*	(0.127)	1.311*	1.056–1.629	0.116	(0.114)
Anorexia	1.022	0.613–1.704	−0.027	(0.234)	0.826	0.488–1.398	0.120	(0.287)
White collar	1.467**	1.127–1.910	−0.305	(0.172)	1.353**	1.111–1.647	−0.382***	(0.109)
Married	1.052	0.833–1.328	−0.367**	(0.147)	0.903	0.750–1.089	−0.435***	(0.103)
ln alpha	0.577	*z-value* *7.74*			0.705	*z-value* *10.29*		
alpha	1.780				2.024			
N (total)	2426				2623			
N (sick days > 0)			507				746	

*OR* odds ratio, *CI* confidence interval, *GPA* grade point average, *SE* standard error

* *p* < 0.05, ** *p* < 0.01, *** *p* < 0.001

### Income

Compared to CG, both males (123,410€ for AN and 156,050€ for CG) and females (109,000€ for AN and 125,030€ for CG) with AN had lower cumulative income in 2011–2016 (Table 1).

AN was associated with lower cumulative income also in the two-part regression model (Table 4). Compared to CG, males with AN were 61% less likely to be working in 2011–2016. On average, working males with AN earned 10% less than working males in the CG. Working females with AN had 12% lower income than working females in the CG in 2011–2016. Females with AN were also less likely to be working compared to the CG, but this difference was not statistically significant.

**Table 4:** Two-part regression model, working status and cumulative income in 2011–2016

	Male				Female			
	Logit		OLS		Logit		OLS	
*Variable*	*OR*	*95% CI*	*Coefficient*	*SE*	*OR*	*95% CI*	*Coefficient*	*SE*
GPA	0.648***	0.552–0.760	−0.007	(0.010)	0.798**	0.683–0.932	0.047***	(0.009)
Lower tertiary education	0.924	0.629–1.356	−0.045	(0.025)	2.008***	1.430–2.820	0.051**	(0.018)
Higher tertiary education	1.361	0.798–2.318	0.038	(0.033)	2.367***	1.518–3.689	0.180***	(0.025)
Parental secondary education	1.064	0.612–1.850	−0.003	(0.036)	0.627	0.331–1.187	0.027	(0.030)
Parental tertiary education	0.630	0.343–1.158	−0.022	(0.042)	0.353**	0.177–0.703	−0.001	(0.035)
Both parents	1.162	0.847–1.593	0.056**	(0.021)	0.874	0.645–1.184	−0.001	(0.017)
Anorexia	0.392**	0.230–0.669	−0.096*	(0.043)	0.565	0.277–1.152	−0.119**	(0.039)
White collar	6.156***	4.169–9.090	0.089***	(0.022)	6.560***	4.911–8.762	0.203***	(0.016)
Married	5.329***	3.406–8.336	0.156***	(0.017)	1.198	0.922–1.556	−0.034*	(0.014)
N (total)	2426				2623			
N (working 2011–2016)			2148				2307	
Pseudo R-squared/R-squared	0.135		0.059		0.146		0.188	

*OLS* ordinary least squares, *OR* odds ratio, *CI* confidence interval, *SE* standard error, *GPA* grade point average

* *p* < 0.05, ** *p* < 0.01, *** *p* < 0.001

## Discussion

### Main findings

To our knowledge, this is the first study to comprehensively explore the current long-term labour market outcomes of AN in terms of unemployment, sickness benefits, and income. Contrary to previous studies that have reported promising labour market outcomes for individuals with AN, our findings indicate that AN is associated with somewhat unfavourable outcomes, particularly in terms of income accumulation. Compared to the CG, AN was associated with lower cumulative income among both males and females between the ages of 25 and 30. For both sexes, AN was associated with a greater number of unemployment days between the ages of 25 and 33 among individuals who experienced any unemployment. Regarding sick days, there were no statistically significant differences between individuals with AN and the CG in either sex.

### Comparison to previous studies

Our finding regarding the association between AN and lower incomes contrasts with a previous Finnish study [7] and Swedish studies [5, 6], which have suggested relatively favourable long-term labour market outcomes in AN regarding employment and receipt of sickness benefits. However, none of these previous studies [5–7], has specifically addressed the association between AN and income. In a broader context, our results extend the existing evidence from Finnish register studies on the adverse socioeconomic consequences of mental disorders—such as reduced income and occupational status [18, 19]—to encompass AN. Due to intensive treatment in adolescence, previous research has linked AN to a higher likelihood of prolonged studies and delayed entry into the workforce [5–7]. Consistent with these earlier findings [5–7], compared to the CG, individuals with AN had lower educational levels at the age of 30 in our study. Our findings regarding reduced income among individuals with AN may be explained by the fact that prolonged studies, due to illness or potential postgraduate education, lead to delayed entry into the workforce, resulting in income loss in early adulthood. Moreover, a higher number of unemployment days among individuals with AN naturally leads to greater income loss compared to the CG.

AN was associated with lower income regardless of sex, but especially among males. Several explanations may account for this sex difference. Firstly, societal weight and beauty standards differ between males and females, which can contribute to income disparities. According to social comparison theory, females are more likely to be rewarded for meeting societal beauty standards, including low weight and thinness [35], potentially leading to higher income [36]. In contrast, males often aim for a more muscular body composition [35], and males with AN are less likely to meet traditional masculine ideals and face more stigma [17]. This may contribute to underdiagnosis and marginalization of males suffering from AN, which lowers their income levels in the long term. Secondly, the gender wage gap leads to greater variation in males’ wages overall compared to females [14]. In our study as well, the variation in males’ wages was greater, which highlighted the income gap between AN and the CG in males. Thirdly, compared to females, more males enter the labour force at an earlier age, while females tend to pursue higher education than males [37], which is also evident in our sample. As a result, wage variation is more pronounced among males in young adulthood, whereas for females, income differences are likely to become more apparent at a later age.

We found that individuals with AN, especially males, had more unemployment days compared to the CG. Additionally, among both males and females who experienced unemployment, those with AN had a higher expected number of unemployment days compared to the CG in the regression model. These findings differ from a previous Finnish study [7], which reported no significant difference in unemployment or disability pensions between females with AN and their unaffected peers. In contrast to this previous study, our research used register data rather than self-reports, and our sample included males. Our previous research indicated that males with undiagnosed mental health disorders are particularly at risk of being excluded from the workforce [19]. Our findings suggest that this conclusion may also be applicable to males with undiagnosed AN. We did not find a significant difference in the mean number of sick days between AN and the CG. Our results align with a previous study, which reported that after a 10-year follow-up, AN was not associated with a higher number of sickness absences in the past year compared to unaffected peers [7]. Together with our other findings, these results reflect that individuals with AN have not yet become fully integrated into the workforce at ages 25–33, which keeps the number of sick days low but leads to more unemployment days and income losses compared to the CG. Our findings expand previous understandings of the recovery prognosis of AN [1] by broadening the focus to the occupational domain and demonstrating the negative long-term impact of AN on labour market outcomes.

Compared to previous national and international studies [11]– [12], the prevalence of AN in our study was higher, especially in males. This is mainly due to our use of a broader definition of AN, in line with previous research highlighting the underdiagnosis of the disorder [11, 12, 16]. Underdiagnosis has been reported to be especially common among males [16, 17]. Previous Finnish studies have shown that different diagnostic criteria can significantly affect the prevalence of AN, and that broader definitions are associated with a better prognosis [38, 39]. However, contrary to these findings, our study found that AN was associated with poorer occupational outcomes, despite using a broader definition of AN. Our finding of a poorer outcome compared to previous studies may be related to the inclusion of males in our sample. However, some studies have suggested that males with AN have a better prognosis than females [40].

### Strengths and limitations

In order to account for current diagnostic practices, changes in working life, and the diagnostic criteria of AN that remain insufficiently inclusive of sex diversity, the general population sample from the NFBC1986, with over 30 years of lifetime follow-up, provides a comprehensive view of the long-term labour market outcomes of AN in the present day. Whereas previous studies have assessed labour market outcomes cross-sectionally using questionnaire-based data on single outcome variables, we deepened our investigation by analysing prospectively collected register data on unemployment, sickness absences, and income to examine how individuals with AN develop their careers and adapt to the labour market. Our sex-specific analyses enabled us to demonstrate differences in labour market outcomes between females and males, considering previous studies that have exclusively examined the labour market outcomes of AN in females [5–7]. By adjusting for factors from childhood to adulthood, we were able to minimise the effects of potential confounding factors.

To account for the apparent underdiagnosis in a general population dataset [12] and the challenges in identifying individuals with atypical symptom profiles [10, 16], we defined AN more broadly than in many previous studies. Therefore, our study sample encompasses a wider range of severity levels, including individuals with sub-threshold AN. Our results cannot be directly generalized or compared to samples consisting solely of individuals who have been hospitalized, as our results represent a general population dataset that also includes individuals in outpatient care and those who remain undiagnosed. However, even though our definition was broader, we still found associations between AN and unfavourable labour market outcomes, highlighting the need for interventions regardless of diagnoses made by physicians. The small sample sizes in some variables limit the generalizability and reliability of the results, particularly regarding the impact of higher parental education and participants’ own educational level among females with AN. Moreover, we could not account for the role of other psychiatric or somatic morbidity, which may affect the outcomes [16]. Our findings are based on a Nordic welfare state, which ensures access to education, healthcare, and social security for all citizens [19]. Therefore, our estimates can be viewed as a lower bound of the true effect.

### Clinical implications

Current approaches to eating disorders are limited in scope and fail to account for their diverse and multifaceted nature, leading to gaps in healthcare practices—particularly in relation to males [16]. Due to non-help-seeking behaviour, males are exposed to the adverse effects of undiagnosed and untreated eating disorders [17, 19]. Eating disorder screening questionnaires have been reported to primarily identify young women with AN [41]. Our results indicate that more effective methods are needed for screening males with AN symptoms in primary, student, and occupational healthcare, as they may not be adequately recognized in the current health care system. In addition to healthcare, awareness among employers on how to address eating disorder symptoms should be improved to ensure adequate support for employees. For example, the recent EU-level guidance on best practices for supporting mental health in the workplace aims to provide practical tools for local interventions, including those related to eating disorders [42].

Our results suggest that young people, especially males, with AN need more effective interventions to improve their labour market outcomes. Due to recent reductions in psychiatric healthcare resources in Finland [9, 10] and in other countries with similar healthcare systems, cost-effective solutions are necessary. For example, the involvement of dietitians should be considered not only in specialized healthcare but especially in primary healthcare [43] and occupational health services, where the diagnosis of eating disorder symptoms and interventions to maintain work ability in working-age individuals typically take place. Additionally, individuals with AN are often in an important transition phase where their healthcare services lie at the intersection of primary healthcare, adolescent psychiatry, adult psychiatry, and occupational healthcare [10]. This may lead to a longer duration of untreated disorder and thus, to worse later outcomes [44]. Based on our results, collaboration between different healthcare providers and unemployment services should be strengthened to ensure early interventions for individuals with AN.

In the future, it is important to investigate the association between AN and labour market outcomes in other countries to enhance the generalizability of our findings. Additionally, the role of other mental disorders and somatic illnesses could be explored. Finally, there is a need for increased awareness of AN in males.

## Conclusion

This study highlights that AN is associated with relatively unfavourable labour market outcomes, particularly in terms of income accumulation. Since the poor outcome is more evident in males, more effective strategies are needed to identify and support males with potential AN. Increasing awareness of AN among students and in workplaces, strengthening collaboration between healthcare providers, unemployment services, and employers, as well as implementing early and cost-effective interventions, are crucial for improving labour market outcomes in AN.

## Supplementary Information

Below is the link to the electronic supplementary material.

Supplementary Material 1

## Data Availability

NFBC data is available from the University of Oulu, Infrastructure for Population Studies. Permission to use the data can be applied for research purposes via electronic material request portal. In the use of data, we follow the EU general data protection regulation (679/2016) and Finnish Data Protection Act. The use of personal data is based on cohort participants written informed consent at their latest follow-up study, which may cause limitations to its use. Please, contact the NFBC project center (NFBCprojectcenter@oulu.f) and visit the cohort website (www.oulu.f/nfbc) for further information.

## References

[1] Solmi M, Monaco F, Højlund M et al (2024) Outcomes in people with eating disorders: a transdiagnostic and disorder-specific systematic review, meta-analysis and multivariable meta-regression analysis. World Psychiatry 23:124–138. 10.1002/wps.21182 38214616 PMC10785991

[2] Richmond TK, Woolverton GA, Mammel K et al (2020) How do you define recovery? A qualitative study of patients with eating disorders, their parents, and clinicians. Int J Eat Disord 53:1209–1218. 10.1002/eat.23294 32453448

[3] Jääskeläinen E, Juola P, Hirvonen N et al (2013) A systematic review and meta-analysis of recovery in schizophrenia. Schizophr Bull 39:1296–1306. 10.1093/schbul/sbs130 23172003 PMC3796077

[4] Molstrom I-M, Nordgaard J, Urfer-Parnas A et al (2022) The prognosis of schizophrenia: a systematic review and meta-analysis with meta-regression of 20-year follow-up studies. Schizophr Res 250:152–163. 10.1016/j.schres.2022.11.010 36417817

[5] Hjern A, Lindberg L, Lindblad F (2006) Outcome and prognostic factors for adolescent female in-patients with anorexia nervosa: 9- to 4-year follow-up. Br J Psychiatry 189:428–432. 10.1192/bjp.bp.105.018820 17077433

[6] Wentz E, Gillberg IC, Anckarsäter H et al (2009) Adolescent-onset anorexia nervosa: 18-year outcome. Br J Psychiatry 194:168–174. 10.1192/bjp.bp.107.048686 19182181

[7] Mustelin L, Raevuori A, Bulik CM et al (2015) Long-term outcome in anorexia nervosa in the community. Int J Eat Disord 48:851–859. 10.1002/eat.22415 26059099

[8] European Union Agency for Safety and Health at Work (EU-OSHA) (2023) Workers with mental disorders in a digitalised world: challenges, opportunities and needs. https://osha.europa.eu/en/publications/workers-mental-disorders-digitalised-world-challenges-opportunities-and-needs

[9] Jahangiri E, Kannisto G, Hakko H, Riipinen P, Räsänen S (2022) Assisted living in relation to use of psychiatric inpatient and outpatient care – a 23-year time-trend analysis of national indicators from Finland. Psychiatr Fenn 53:190–203

[10] Silén Y, Rosenqvist A, Aalto-Setälä T, Suvisaari J, Linnanranta O (2025) Hyvinvointialueille kohdennettu selvitys syömishäiriöiden palveluista: osaamista ja yhteistyötä tulee vahvistaa. THL – Työpaperi 6/2025. https://urn.fi/URN:ISBN:978-952-408-456-7

[11] Qian J, Wu Y, Liu F et al (2022) An update on the prevalence of eating disorders in the general population: a systematic review and meta-analysis. Eat Weight Disord 27:415–428. 10.1007/s40519-021-01162-z 33834377 PMC8933366

[12] Keski-Rahkonen A, Hoek HW, Susser ES et al (2007) Epidemiology and course of anorexia nervosa in the community. Am J Psychiatry 164:1259–1265. 10.1176/appi.ajp.2007.06081388 17671290

[13] Campbell OLK, Bann D, Patalay P (2021) The gender gap in adolescent mental health: a cross-national investigation of 566,829 adolescents across 73 countries. SSM 13:100742. 10.1016/j.ssmph.2021.100742 PMC7960541 33748389

[14] International Labour Office (ILO) World Employment and Social Outlook: Trends for women 2017. https://www.ilo.org/publications/world-employment-and-social-outlook-trends-women-2017

[15] McMunn A, Lacey R, Worts D et al (2015) De-standardization and gender convergence in work–family life courses in great britain: a multi-channel sequence analysis. Adv Life Course Res 26:60–75. 10.1016/j.alcr.2015.06.002

[16] Prashar E, Schulze KJ, Furlano JA (2025) Addressing disparities in eating disorders: underfunding, research gaps and clinical training deficiencies among males and men. Br J Psychiatry 1–3. 10.1192/bjp.2025.70 40190175

[17] Gorrell S, Murray SB (2019) Eating disorders in males. Child Adolesc Psychiatr Clin N Am 28:641–651. 10.1016/j.chc.2019.05.012 31443881 PMC6785984

[18] Hakulinen C, Böckerman P, Pulkki-Råback L et al (2021) Employment and earnings trajectories before and after sickness absence due to major depressive disorder: a nationwide case–control study. Occup Environ Med 78:173–178. 10.1136/oemed-2020-106660 33051385

[19] Majuri T, Huikari S, Jääskeläinen E et al (2024) Mental disorder symptoms and diagnoses are differently associated with labour market attachment and registered income until midlife: the Northern Finland birth cohort 1966. Int J Soc Psychiatry 00207640241299384. 10.1177/00207640241299384 PMC12171070 39601426

[20] University of Oulu (1986) Northern Finland Birth Cohort 1986. http://urn.fi/urn:nbn:fi:att:f5c10eef-3d25-4bd0-beb8-f2d59df95b8e). Accessed 20 Jan 2025

[21] Järvelin MR, Elliott P, Kleinschmidt I et al (1997) Ecological and individual predictors of birthweight in a Northern Finland birth cohort 1986. Paediatr Perinat Epidemiol 11:298–312. 10.1111/j.1365-3016.1997.tb00007.x 9246691

[22] Silén Y, Sipilä PN, Raevuori A et al (2020) DSM-5 eating disorders among adolescents and young adults in finland: A public health concern. Int J Eat Disord 53:520–531. 10.1002/eat.23236 31999001

[23] Statistics Finland (2025) Classifications. https://stat.fi/en/luokitukset. Accessed 15 Jan 2025

[24] International Standard Classification of Education (ISCED) (2011) (https://ec.europa.eu/eurostat/statistics-explained/index.php/International_Standard_Classification_of_Education_(ISCED)). Accessed 15 Sep 2024

[25] Studyinfo (2025) The register of the Finnish national application system for upper secondary education. https://opintopolku.fi/konfo/en/. Accessed 20 Jan 2025

[26] Digital and Population Data Services Agency (2025) Population Information System. https://dvv.fi/en/population-information-system. Accessed 20 Jan 2025

[27] Finnish Centre for Pensions (2025) Statistics. https://www.etk.fi/en/research-statistics-and-projections/statistics/. Accessed 15 Sep 2024

[28] The Social Insurance Institution of Finland (2025) Statistics. https://www.kela.fi/web/en/statistics. Accessed 15 Sep 2024

[29] The Finnish Tax Administration (2025) Statistics. https://www.vero.fi/en. Accessed 15 Sep 2024

[30] Statistics Finland (2023) Employment. https://stat.fi/en/statistics/tyokay. Accessed 15 Jan 2025

[31] Finnish Immigration Service (2024) Income requirement. https://migri.fi/en/income-requirement. Accessed 15 Jan 2025

[32] Tse S, Chan S, Ng KL, Yatham LN (2014) Meta-analysis of predictors of favorable employment outcomes among individuals with bipolar disorder. Bipolar Disord 16:217–229. 10.1111/bdi.12148 24219657

[33] Díaz-Caneja CM, Pina-Camacho L, Rodríguez-Quiroga A et al (2015) Predictors of outcome in early-onset psychosis: a systematic review. NPJ Schizophr 1(1):14005. 10.1038/npjschz.2014.5 27336027 PMC4849440

[34] Ajnakina O, Stubbs B, Francis E et al (2021) Employment and relationship outcomes in first-episode psychosis: a systematic review and meta-analysis of longitudinal studies. Schizophr Res 231:122–133. 10.1016/j.schres.2021.03.013 33839370

[35] Murray SB, Nagata JM, Griffiths S et al (2017) The enigma of male eating disorders: a critical review and synthesis. Clin Psychol Rev 57:1–11. 10.1016/j.cpr.2017.08.001 28800416

[36] Edwards CH, Bjørngaard JH, Minet Kinge J (2021) The relationship between body mass index and income: using genetic variants from HUNT as instrumental variables. Health Econ 30:1933–1949. 10.1002/hec.4285 33993584

[37] Ek E, Ala-Mursula L, Velázquez RG et al (2021) Employment trajectories until midlife associate with early social role investments and current work-related well-being. Adv Life Course Res 47:100391. 10.1016/j.alcr.2020.100391 36695148

[38] Mustelin L, Silén Y, Raevuori A et al (2016) The DSM-5 diagnostic criteria for anorexia nervosa may change its population prevalence and prognostic value. J Psychiatr Res 77:85–91. 10.1016/j.jpsychires.2016.03.003 27014849

[39] Silén Y, Raevuori A, Jüriloo E et al (2015) Typical versus atypical anorexia nervosa among adolescents: clinical characteristics and implications for ICD-11. Eur Eat Disord Rev 23(5):345–351. 10.1002/erv.2370 26010207

[40] Strobel C, Quadflieg N, Naab S et al (2019) Long-term outcomes in treated males with anorexia nervosa and bulimia nervosa-a prospective, gender-matched study. Int J Eat Disord 52:1353–1364. 10.1002/eat.23151 31444805

[41] Kutz AM, Marsh AG, Gunderson CG et al (2020) Eating disorder screening: a systematic review and meta-analysis of diagnostic test characteristics of the SCOFF. J Gen Intern Med 35:885–893. 10.1007/s11606-019-05478-6 31705473 PMC7080881

[42] European Union Agency for Safety and Health at Work (EU-OSHA) (2024) Guidance for workplaces on how to support individuals experiencing mental health problems. https://osha.europa.eu/en/publications/guidance-workplaces-how-support-individuals-experiencing-mental-health-problems

[43] Howatson A, Wall CR, Turner-Benny P (2015) The contribution of dietitians to the primary health care workforce. J Prim Health Care 7:324–332. 10.1071/hc15324 26668838

[44] Austin A, Flynn M, Richards K et al (2021) Duration of untreated eating disorder and relationship to outcomes: a systematic review of the literature. Eur Eat Disord Rev 29:329–345. 10.1002/erv.2745 32578311

