# Supplementary material for: Long-term labour market outcomes of anorexia nervosa – the Northern Finland birth cohort 1986
Source: Soc Psychiatry Psychiatr Epidemiol. 2025 Aug 26;61(10):2037–46. doi: 10.1007/s00127-025-02983-4 (PMC13585820; doi:10.1007/s00127-025-02983-4)
Supplement: Supplementary file 1 [file 127_2025_2983_MOESM1_ESM.docx]

Long-term labour market outcomes of anorexia nervosa – the Northern Finland Birth Cohort 1986

Tuomas Majuri^1^ · Emmi Wilén · Sanna Huikari · Marko Korhonen

^1^Research Unit of Population Health, University of Oulu, Oulu, Finland.

Corresponding author:

M.D. Tuomas Majuri,

email tuomas.majuri@student.oulu.fi

**Online supplement table 1.** Characteristics of the sample (n=5049)

| Variable |  | Male | |  | Female | |
| --- | --- | --- | --- | --- | --- | --- |
|  |  | Anorexia (n=92) | Control group (n=2334) |  | Anorexia (n=73) | Control group (n=2550) |
| **Parents’ educational level, n (%)** |  |  |  |  |  |  |
| Basic |  |  | 141 (6.0) |  | 5 (6.9) | 132 (5.2) |
| Secondary |  |  | 1729 (74.1) |  | 59 (80.8) | 1936 (75.9) |
| Tertiary |  | 13 (14.1) | 464 (19.9) |  | 9 (12.3) | 482 (18.9) |
| Basic/secondary* |  | 79 (85.9) |  |  |  |  |
| **Educational level, n (%)** |  |  |  |  |  |  |
| Basic/secondary |  | 65 (70.7) | 1394 (59.7) |  | 32 (43.8) | 1073 (42.1) |
| Lower tertiary |  |  | 556 (23.8) |  | 31 (42.5) | 902 (35.4) |
| Higher tertiary |  |  | 384 (16.5) |  | 10 (13.7) | 575 (22.6) |
| Tertiary* |  | 27 (29.4) |  |  |  |  |
| **Marital status, n (%)** |  |  |  |  |  |  |
| Married/registered partnership |  | 25 (27.2) | 702 (30.1) |  | 31 (42.5) | 1043 (40.9) |
| Not married/not registered |  | 67 (72.8) | 1632 (69.9) |  | 42 (57.5) | 1507 (59.1) |
| **Family type, n (%)** |  |  |  |  |  |  |
| Both parents |  | 70 (76.1) | 1881 (80.6) |  | 58 (79.5) | 1996 (78.3) |
| Others |  | 22 (23.9) | 453 (19.4) |  | 15 (20.6) | 554 (21.7) |
| **Socioeconomic status, n (%)** |  |  |  |  |  |  |
| White collar |  | 31 (33.7) | 1000 (42.8) |  | 54 (74.0) | 1625 (63.7) |
| Other |  | 61 (66.3) | 1334 (57.2) |  | 19 (26.0) | 925 (36.3) |
| **GPA, M (SD)** |  | 7.31 (1.03) | 7.46 (1.06) |  | 7.88 (0.98) | 8.03 (1.00) |

*GPA* grade point average*, M* mean, *SD* standard deviation

*Note: * = Categories with a low number of observations (<5) are combined with the neighbouring category to ensure anonymity and to comply with the guidelines set by the Finnish Social and Health Data Permit Authority, Findata (https://findata.fi/en/services-and-instructions/producing-anonymous-results/).*
